# Investigating the role of the general practitioner in cancer prevention: a mixed methods study

**DOI:** 10.1186/1471-2296-14-58

**Published:** 2013-05-07

**Authors:** Sonja McIlfatrick, Sinead Keeney, Hugh McKenna, Nigel McCarley, Gerry McElwee

**Affiliations:** 1 Reader, Institute of Nursing and Health Research, University of Ulster, Shore Road, Newtownabbey, N. Ireland, BT37 OQB, UK; 2 Senior Lecturer, Institute of Nursing Research and Health, University of Ulster, Ulster, N. IrelandUK; 3 Prof Hugh McKenna, Pro Vice Chancellor Research & Innovation, University of Ulster, Ulster, N. Ireland, UK; 4 Dr Nigel McCarley, Research Associate, Institute of Nursing and Health Research, University of Ulster, Ulster, N. Ireland, UK; 5 McIlwee, G Head of Cancer Prevention, Cancer Focus NI, Ulster, N. Ireland, UK

**Keywords:** Cancer, Prevention, General practitioners, Primary health care, Health promotion, Mixed methods

## Abstract

**Background:**

Despite evidence of the effectiveness of cancer preventive services and the increasing development of guidelines, actual rates of delivery of cancer prevention activities remain low. Due to their frequent front-line contact with the public, family physicians (GPs) have the potential to play an important role in the primary prevention of cancer. However, there is a lack of information about their actual role in cancer prevention. The aim of this study was to investigate the actual and potential roles of general practitioners (GP) in the prevention of cancer.

**Methods:**

A sequential exploratory mixed methods approach was used. The sample included all the General Practice (GP) practices in a region in the UK (n=345). Postal questionnaires were administered to GPs (n=1249); following 290 returns (response rate 23%), semi-structured interviews were undertaken with GPs (n=14).

**Results:**

The majority of the GP respondents (66.4%, n=184) considered that they routinely provided cancer prevention information. This was specifically focusing on smoking cessation as almost all GPs (96.8%, n=270) enquired about a patient’s smoking status. Overall, 47.2% (n=128) of GP respondents indicated that they felt they did not have time to perform a cancer prevention role; however, 88.3% (n=242) still felt that they had the ‘opportunity’ to do so. Over half the sample (61.3%, n=168) indicated that imposed health priorities and targets militated against providing cancer prevention activities. Almost all the GP respondents (98.9%, n=273) agreed with empowering individuals to take responsibility for their health issues. The GPs identified the need for alternative models for cancer prevention beyond current face to face patient care, including other health and non-health professionals. Whilst lack of time was identified as a critical factor, the GPs indicated that significant efforts were made to encourage patients to take personal responsibility for lifestyle choices.

**Conclusions:**

The GPs indicated a need for training around behavioural change and theories of motivation and action. This has implications for primary care and family physicians worldwide. While doctor–patient consultations and the physicians’ credibility offer great potential for cancer prevention, time pressures and imposed government targets often mean that their actual cancer prevention role is reduced.

## Background

Cancer remains a major cause of disease worldwide and according to the World Health Organisation (WHO) will result in 12 million deaths by 2030 [1]. In general, cancer prevention activities include avoiding risk factors such as smoking and increasing protective factors such as eating a healthy diet [2]. According to the National Cancer Institute [3], 80% of all cancers are due to identifiable factors and as such are potentially preventable. It has been suggested that prevention offers the most cost-effective long-term strategy for the control of cancer worldwide [4]. The European Code against Cancer [5] identified key behaviours that, if modified, will lead to both a reduction in cancers and improvement in general health (See Section List). The key risk factors to avoid cancer are widely identified as the use of tobacco; being overweight and obese; poor diet; physical inactivity; the harmful use of alcohol, sexually transmitted human papilloma Virus (HPV) occupational hazards and exposure to UV radiation [6-11]. Lutfiyya *et al.*[12] suggested that obesity was rapidly approaching tobacco as the leading cause of preventable morbidity and mortality. Danaei *et al.*[8] analysed data from seven million cancer deaths worldwide and estimated that 35% of cancer deaths were attributable to nine potentially modifiable behavioural and environmental risk factors. Much of the modifiable risk factor avoidance and reduction in cancer prevention have been centred on lifestyle issues and behavioural change [13,14]. Cancer prevention, according to the National Cancer Institute (NCI), is both the promotion of healthy behaviours as well as the avoidance of risk factor behaviours. These concepts are inherent in health promotion theory and practice [14,15]. It is evident that both health promotion and disease prevention are not two discreet entities but there is a significant degree of overlap between them [16].

Section List: **European Code against Cancer (2003)**

Healthier Lifestyle Factors

1. Do not smoke.

2. Avoid Obesity.

3. Undertake some brisk, physical activity every day.

4. Increase your daily intake and variety of vegetables and fruits: eat at least five servings daily.

5. Moderate consumption of alcohol: to two drinks per day if you are a man and one drink per day if you are a woman.

6. Care must be taken to avoid excessive sun exposure.

7. Apply strictly regulations aimed at preventing any exposure to known cancer causing substances.

Public health programmes

8. Cervical screening - women from 25 years of age.

9. Breast screening -women from 50 years of age.

10. Colorectal screening - men and women from 50 years of age.

11. Vaccination programmes against Hepatitis B Virus infection.

It has been suggested that due to their frequent contact with the public, GPs could play an important role in primary and secondary (screening) cancer prevention [17]. There is, however, a limited amount of empirical research on cancer prevention in primary care. Previous research about the role of primary care physicians in prevention and health promotion has been concentrated on specific topics such as attitudes to and involvement in health promotion and lifestyle counselling [18] and perception of GPs in modifying behaviour [19]. More recently Broton et al. (on behalf of the EUROPREV network) [20] undertook a large multinational study across eleven European countries (n=2082 GPS) seeking the views of GPs on prevention and health promotion in clinical practice. They found that although GPs believed they should advise preventive and health promotion activities, in practice, they were less likely to do so, reporting heavy workload/lack of time and no reimbursement as the main barriers.

Austoker [13] identified nine specific barriers to GPs participation in prevention activities. These included aspects such as a lack of motivation, training, time, support, appropriate health education resources and protocols; concerns with low success rates; inadequate financial reimbursement; and a failure to use the skills of other members of the primary care team. The issue of time constraints was examined further in a study in the US, which sought to determine the amount of time required for a primary care physician (GP) to provide recommended preventive services (as per the US Preventive Services Task Force (USPSTF), to an average patient panel [21]. They found that in order to fully satisfy the USPSTF recommendations, 7.4 hours per working day, was needed for the provision of preventive services. These authors contended that the amount of time required is overwhelming; suggesting that primary care physicians cannot achieve preventive services goals unassisted.

It has been noted that patients with cancer in the United Kingdom tend to present with more advanced disease and have poorer survival rates than many of their European counterparts [22,23]. The most likely explanations for this are either late presentation by patients or late onward referral by general practitioners. Two systematic literature reviews [24,25], investigating risk factors for patient delay in presenting with common cancers have shown the predominant risk factors to be lack of awareness of the seriousness of the symptom or not recognising that the symptom could be caused by cancer. More recently, a population based survey of public awareness of cancer in the UK, [26] showed that awareness was lower in males, younger adults, persons from lower socioeconomic groups and among ethnic minorities. A study carried out in Northern Ireland into the knowledge, attitudes and behaviours of people in mid-life to cancer prevention; found that participants felt that the GP should be more pro-active in the prevention of cancer, primarily through the provision of both verbal and written information [27]. Such evidence highlights the importance of examining the actual and potential role of GP in cancer prevention so as to help inform the future development of targeted cancer prevention strategies. The aim of this study was to investigate the actual and potential roles of the GP in the prevention of cancer.

## Methods

The study used a sequential exploratory mixed methods approach. This was undertaken in two methodological stages: stage one a cross-sectional questionnaire survey and stage two exploratory one to one interviews.

### Participants

Data indicated that there were a total of 364 General Practices within Northern Ireland, with a total of 1,168 GPs employed [28]. As this data did not include locum GPs, each practice manager was contacted to confirm the number of GPs and was sent a Practice Proforma (detailing generic practice data); and Questionnaires for GPs. This resulted in a total of 1,249 questionnaires distributed. Two rounds of follow up phone calls were made to each practice to enhance response rate.

### Data collection

The questionnaire was developed to reflect a risk factor-oriented approach. Therefore, elements of the European Code against Cancer [5] were used as its framework. This included primarily closed response questions; response option questions, Likert scales and the inclusion of a free text ‘other’ category (Additional file 1). The survey respondents were asked to return a postcard independently of their completed questionnaires if they wished to take part in a follow up individual interviews. Of those who did this, fourteen participants were purposively selected for interview, allowing geographic spread (n=14). The interviews were conducted by the research fellow for the study (NMc). Emergent themes from the analysis of the stage one survey informed the interview schedule. The interview questions in stage two, focused on exploring how the participants felt the role of the GP in cancer prevention could be further developed.

### Data analysis

Quantitative data were analysed using SPSS employing descriptive and non-parametric statistics. Qualitative data were recorded, transcribed and content analysed using the three-stage approach to data analysis suggested by Strauss and Corbin [12].

### Ethical considerations

The stage one survey assured anonymity and confidentiality. However, anonymity was sacrificed for those respondents who volunteered to be interviewed in stage two, because of returning the postcard independently of their completed questionnaires their stage one responses remained confidential. Ethical approval was obtained from the Office for Research Ethics for Northern Ireland (ORECNI) prior to the beginning of the study.

## Results

Two hundred and ninety questionnaires were returned. This constituted a representative sample response rate of 23% with a confidence level of 95% and an interval of 5.06.

### Demographics

Respondents were predominately male (55.6%, n=154) worked full time (72.3%, n=201) and were principal GPs (90.9%; n=250). Furthermore, 26.1% (n=72) of GPs who completed the Stage 1 questionnaire had a lead responsibility for cancer services within the practice and 20.8% (n=57) had completed a post-graduate course in cancer prevention/treatment (Table 1).

**Table 1:** Demographic details

	**GP**	
	**%**	**N**
Female	44.4%	123
Male	55.6%	154
Full time	72.3%	201
Part-time	27.7%	77
Principal GP	90.9%	250
Salaried GP	3.3%	9
Retained GP	0.4%	1
Locum GP	5.5%	15

N=277.

Stated returns were 290 with 8 not being able to be used- 282.

### Actual role in cancer prevention

**Figure 1:**
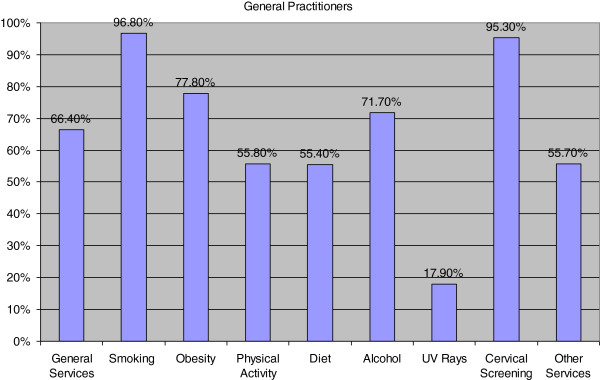
The provision of cancer prevention activities as stated by GPs.

Each participant was asked to indicate what services they provided and how often they would typically do this. Nine broad categories (incorporating the elements of the European Code against Cancer) were identified as important in cancer prevention. These were ‘general services relating to smoking; obesity; physical activity; diet; alcohol; exposure to UV rays; cervical screening; and ‘other services’. Figure 1 highlights the percentage of services GPs would routinely provide for their patients. In general, GPs tended to focus mostly on the smoking behaviour of patients and the provision of cervical screening with a small input into advice in relation to UV exposure (17.9%). It was found that most GPs respondents (66.4%, n=184) considered that they routinely practised cancer prevention, with 31.8% of respondents routinely providing written information.

This position was supported from the analysis of stage two qualitative data and is reflected in the following comment:

*“We see people here running with four or five chronic illnesses, do you know. Maybe they’re not thinking of cancer. They’re thinking of keeping everything else on, you know, you have your COPD, then the diabetic and they’re overweight, and as you say, they’re anxious, they’re depressed. They want to go to counselling, you know. It’s prioritising a lot of that, and I suppose cancer prevention drops down when you have a lot of co-morbidity”* (GP6)

### The provision of cancer avoidance services

The results from the questionnaire showed that most cancer prevention activities that take place in primary care address many of the risk factors associated with maintaining a healthy lifestyle. For example, when explicitly managing general patient issues around obesity, diet, alcohol consumption, physical exercise and smoking behaviours, the principal risk factors for cancer are also being addressed implicitly.

#### Services related to smoking

Almost all GPs (96.8%, n=270) enquired about a patient’s smoking status. To reduce patients’ smoking 86.6% (n=240) reported routinely providing pharmacological help, 64.3% (n=176) routinely providing brief advice clinics and specialist support clinics (57.8%, n=159). In keeping with the responses from the Stage 1 questionnaires, the primary intervention specifically aimed at cancer prevention was consistently reported to be related to smoking cessation.

*“The big thing that we would advocate is smoking cessation. That’s the main cancer causing drug as such, so all our patients are actively encouraged to stop smoking”* (GP11)

*“I think it’s also partly driven by our indicators, our QOF – (quality and outcome framework) has built into it, an incentive to establish everybody’s smoking status, if they smoke, establish it every 18 months you have to keep asking them and keep recording them”.* (GP4)

#### Services related to obesity, diet and physical activity

The majority (77.8%; n=217) of the GP respondents routinely measured the BMI of patients. However, 37.3% (n=104) did not provide information on the relationship between obesity and cancer. GP respondents were less engaged in enquiring about a patient’s physical activity levels on a routine basis with only 55.8% (n=154) doing so. Related to this, 51.5% (n=152) did not provide any information linking physical activity and cancer. Overall, 55.4% (n=154) of GP respondents routinely asked about the patient’s diet and notably, 32.1% (n=89) did not provide patients with literature relating to diet and cancer prevention. Lack of demand and/or lack of resources were perceived as the main reason for not engaging in these activities.

It is also of note that patients frequently presented with co-morbidities and this influenced the focus on cancer prevention. As one GP interviewee explained:

*“we see people here running with four or five chronic illnesses, do you know. Maybe there’re not thinking of cancer. They’re thinking of keeping everything else on, you know, you have your COPD, then the diabetic and they’re overweight, and as you say, they’re anxious, they’re depressed. They want to go to counselling, you know. It’s prioritising a lot of that, and I suppose cancer prevention drops down when you have a lot of co-morbidity”* (GP6)

### Services relating to screening

The provision of cervical screening services to women is largely a routine procedure with 95.3% (n=266) of GP respondents promoting it. Overall, 75% (n=210) of GP respondents indicated that they did so routinely and 19.3% (n=54) did so sometimes. The majority of GPs (55.3%) also routinely promoted active screening for cancers other than cervical cancers.

### Barriers & facilitators to cancer prevention role

Overall, 47.2% (n=128) of GP respondents indicated that they felt they did not have time to perform a cancer prevention role; however, 88.3% (n=242) still felt that they had the ‘opportunity’ to do so. Over half the sample (61.3%, n=168) indicated that government imposed health priorities and targets militated against having a cancer prevention role.

GP interviewees indicated that time dictates that their primary role is more focused on treating presenting problems rather than preventing future ill-health. They maintained that cancer prevention activities are unplanned and opportunistic, for example:

“I think the nature of the job is more intervention treatment focussed........I don’t think GPs think preventionally. I think GPs think in terms of treatment interventions. So in other words, prevention isn’t a priority for GPs. But I think they take opportunities that arise, to communicate a prevention message” (GP5)

*“Time, it’s always time. Ten minute slot – people coming in with a whole variety of problems. It’s very hard to allocate that time as well for prevention” ..........“prevention isn’t a priority for GPs. But I think they take opportunities that arise, to communicate a prevention message”.........“we are pro-active, but it’s in an opportunistic way”* (GP6)

Almost all GP respondents (98.9%, n=273) approved of empowering individuals to take responsibility in making decisions regarding health issues with 64.1% (n=177) strongly agreeing to this role. Overall, 98.9% (n=271) agreed that individuals should be provided with information about better lifestyle choices with 88.4% (n=244) stating that this should be coordinated, offering equality of access to all (91.6%, n=252).

### The potential role of GP in the prevention of cancer

In Stage two the respondents were asked to identify areas for further development of their cancer prevention role (Table 2). Most were in agreement that there was a need to develop further the cancer prevention role. Overall, 79.3% (n=218) were supportive of additional inter-professional practice-based training with 72% (n=198) advocating a link into a strategic plan at a practice level. 78.8% (n=215) of GPs stated that other staff could be developed to make a valued contribution to cancer prevention. This included having better links with outside agencies (69.8%; n=192) and developing a more active role for nurses in cancer prevention within the community (71.6%; n=195).

*“They (Practice Nurses) tend to have more time with the patients and they tend to work with patients in an educational role, rather than a GP does”* (GP5)

*“I would see it more maybe that the nurses could take on that (cancer prevention), in some role ........because we (*GPs*) deal with the illnesses, nurses deal with the more preventative and education roles”* (GP9)

**Table 2:** Potential role of GP in cancer prevention

	**Strongly agree**	**Agree**	**No opinion**	**Disagree**	**Strongly disagree**
Empowering individuals to make their own decisions about health issues	64.1% (n=177)	34.8% (n=96)	0.7% (n=2)	0.4% (n=1)	0% (n=0)
Offering advice to inform individuals about better lifestyle choices	66.8% (n=183)	32.1% (n=88)	0.7% (n=2)	0.4% (n=1)	0% (n=0)
Working with local communities to empower them to make decisions about lifestyle choices	22.3% (n=61)	39.9% (n=109)	27.1% (n=74)	8.8% (n=24)	1.8% (n=5)
Ensuring a co-ordinate cancer prevention approach within the practice?	34.4% (n=95)	54.0% (n=149)	9.1% (n=25)	2.5% (n=7)	0% (n=0)
Identifying patients at risk?	54.7% (n=155)	43.1% (n=119)	1.4% (n=4)	0.7% (n=2)	0% (n=0)
Ensuring equality of access to cancer prevention interventions?	51.6% (n=142)	40.0% (n=110)	4.7% (n=13)	1.8% (n=5)	1.8% (n=5)

In essence, UK GPs are business people and over 65% (n=180) of respondents still felt that the lack of financial incentives for cancer prevention was an issue. Most respondents [77.8% (n=214) felt that better online access to cancer prevention resources would help develop their role.

### Perceived responsibility, knowledge and acceptability of a GP cancer prevention role

Almost all GPs surveyed (92.7%; n=256) were very positive about their responsibility for having a cancer prevention role, with 67.4% (n=186) indicating that they had a responsibility to screen high risk cancer groups. Less than half 48.9% (n=134) disagreed that they spend too much time on the treatment of cancer rather than providing cancer prevention interventions. While 74.6% (n=205) indicated that they felt confident to educate patients about cancer prevention, over half (n=65.6%, 181) felt that they themselves required up-to-date information on cancer prevention strategies. In total, 60% (n=165) indicated that they required a better understanding of the process of changing patients’ opinions and behaviours (Table 3).

**Table 3:** Perceived responsibility, knowledge and acceptability of GP cancer prevention role

**Responsibility**	**Strongly agree**	**Agree**	**No opinion**	**Disagree**	**Strongly disagree**
GPs should try and provide cancer prevention	29.3% (n=81)	63.4% (n=175)	3.6% (n=10)	3.6% (n=10)	0% (n=0)
GPs spend too much time on the treatment of cancer rather than providing cancer prevention	4.0% (n=11)	20.8% (n=57)	26.3% (n=72)	45.6% (n=125)	3.3% (n=9)
GPs have a responsibility to screen high-risk cancer groups	14.9% (n=41)	52.5% (n=145)	17.4% (n=48)	12.0% (n=33)	3.3% (n=9)
**Knowledge**	**Strongly agree**	**Agree**	**No opinion**	**Disagree**	**Strongly disagree**
I have sufficient knowledge to educate clients about cancer prevention	11.3% (n=31)	63.3% (n=174)	15.3% (n=42)	9.1% (n=25)	1.1% (n=3)
I require up-to-date information on cancer prevention strategies	10.9% (n=30)	54.7% (n=151)	17.0% (n=47)	16.7% (n=46)	0.7% (n=2)
I require a better understanding of how to change opinions regarding cancer prevention	6.9% (n=19)	53.1% (n=146)	20.7% (n=57)	17.5% (n=48)	1.8% (n=5)
**Perceived Acceptability**	**Strongly agree**	**Agree**	**No opinion**	**Disagree**	**Strongly disagree**
Patients are very set in their ways and do not want to change	4.0% (n=11)	36.3% (n=101)	11.9% (n=33)	46.8% (n=130)	1.1% (n=3)
Patients do not like the GP to meddle in their private life	2.5% (n=7)	16.2% (n=45)	15.5% (n=43)	62.5% (n=173)	3.2% (n=9)
Patients do not approach their GP for advice on cancer prevention	1.8% (n=5)	25.7% (n=71)	8.7% (n=24)	62.0% (n=171)	1.8% (n=5)
GPs may increase anxiety in the patient population by undertaking cancer prevention activities	2.5% (n=7)	32.9% (n=91)	15.5% (n=43)	45.5% (n=125)	4.0% (n=11)
After consultation with a client on cancer risk, I don’t think they will follow my recommendation	2.2% (n=6)	11.2% (n=31)	32.1% (n=89)	51.3% (n=142)	3.2% (n=9)

Results suggested that GPs appear ambivalent as to whether they could alter a patient’s lifestyle, with 40.3% (n=112) agreeing that patient’s behaviours are established and difficult to change. Overall, 63.8% (n=182) believed that patients found them a valuable source of information on to cancer prevention. A sizeable minority (35.4%, n=98) felt that, if they took a proactive approach to cancer prevention, this would cause an increase in patient anxiety. Nevertheless, 54.5% (151) of the sample indicated that if they provided advice on the risk of cancer, such advice would not be followed.

This was supported by comments from the interviews:

*“If they want to change, they will be receptive, but as you know, a lot of people are totally rigid in their way of life and have no interest”............”I think the majority of the patients would respond, you know. I think if you sit and give the patient a bit of time, and you know, take their concerns seriously and respond, I think a majority of patients will respond positively”* (GP3)

*“They still have to have the motivation themselves to do it. But I think it does – if you keep on, it’s like the water dripping on the stone, keep on with it, I think they do listen.* (GP8)

## Discussion

The majority of GPs in this study considered that they had a clear role in cancer prevention, albeit within a wider health promotion agenda, focusing specifically on smoking cessation and cervical screening. The main reason cited for this limitation in their role was time constraints and imposed government targets. Despite this, the GPs in this study also considered that the primary care consultation did provide a good opportunity for cancer prevention activity and that there is potential to develop cancer prevention within this setting, acknowledging the need for alternative models of cancer prevention provision.

Disease prevention and health promotion are recognised tasks in the daily practice of all general practitioners (GPs) [29]. A recent suggested definition of general practice emphasized the role of GPs in prevention, stating that “the general practitioner engages with autonomous individuals across the fields of prevention, diagnosis, cure, care and palliation using and integrating the sciences of biomedicine, medical, psychology and medical sociology” [30]. It can be argued that this indicates a need for not only ensuring that preventive health care is part of everyday practice in primary care but also the importance of developing an increasing understanding of biopsychosocial approaches to health care. This need for a re-orientation towards prevention also fits with the increasing evidence that recommendations from family practitioners can increase substantially the likelihood of patients undertaking preventive activities [31], alongside the view that a lack of such recommendations has been linked with patient noncompliance [32]. Despite this call for a possible reorientation towards prevention, the findings also indicated that GPs considered that they were primarily interventionist rather than preventionist in their clinical practice. The main reason cited was the need to address the patients’ problems at consultation and that the time available limited the opportunity to engage in prevention activities, unless directly linked to the presenting problem.

The findings showed that the principal activities undertaken by GPs and explicitly linked to cancer prevention were smoking cessation and cervical screening. Whilst this finding in relation to smoking cessation, is similar to other studies that have examined both GPs and nurses in primary care [17,33] a significant issue is that caution is needed to ensure this moves beyond merely routinistic practice and that GPs don’t consider such discussions too time-consuming and ineffective [34]. This is vital in light of the evidence, where tobacco use is recognised as the single most important risk factor for cancer [6]. It was also noted that whilst the majority of GPs in this study did address the other important risk factors for cancer e.g. alcohol consumption, obesity, diet and physical exercise, there was a substantial sample of GPs who did not provide information on these factors, despite the importance of these risk factors having been previously identified in the literature [6,8-11]. This is a significant issue as perhaps GPs may not be convinced by the evidence base on these risk factors. For example, Broton et al’s [20] multinational European survey, found that more than half of the GPs were sceptical of helping patients decrease alcohol consumption, achieve or maintain normal weight and practice regular physical exercise. McAvoy [35], however, contended that this scepticism around helping patients’ achieve lifestyle changes could be alleviated following the provision of education, advice and support for general practitioners.

The two main barriers identified for the actual and potential role of the GP in cancer prevention were remuneration and issues related to workload and time. Whilst the importance of these factors are not new and have been previously identified as the most important barriers for overall health promotion in primary care in a World Health Organisation survey of over 2300 GPs in 16 countries [36], of significance is that the evidence base on the importance of remuneration is inadequate. This is further compounded by the complexity of health care systems making international comparisons difficult. In order to try to address this, Dahrouge et al. [37] examined the impact of remuneration and organisational factors on completing preventive activities in primary care settings. They undertook a cross sectional survey to compare the delivery of preventive services by practices (n=137 practice; 288 family physicians) in four different primary care funding models in Canada. They found that no funding model was clearly associated with superior preventive care. Rather factors such as physician characteristics and practice structure were stronger predictors of performance. For example, practices with one or more female physicians, a smaller patient load and an electronic reminder system had superior prevention scores. This raises some questions around the rhetoric of the importance of remuneration as an important barrier for cancer prevention activities in primary care and the need to balance this with other factors such as practice structure and physician characteristics.

It was also noted that whilst the cancer prevention activities performed by GPs were viewed as opportunistic, the GPs considered that the primary care consultation did provide a good opportunity for cancer prevention activity. This reflects international research indicating that that consultations in primary health care are “ideal for health promotion” [38]. Almost all GPs in this study agreed with empowering individuals to take responsibility for making decisions regarding health issues and providing patients with information about better lifestyle choices. While identifying lack of time as a critical limiting factor, the GPs indicated that significant efforts were and should be made to encourage patients to take personal responsibility for lifestyle choices and changing their behaviours. However, the GPs indicated a need for training around behavioural change, specifically on theories of motivation and action. The development of such activities raises questions around alternative models that extend beyond the current model of face to face patient care. These include possibilities around group activities, the use of technology and other forms of information, social media and social network sites may offer significant potential to inform and influence health behaviours in cancer prevention but this remains an area that is underexploited. Furthermore, other alternatives could include developing the role of both clinical and non-clinical professionals (such as health educators and dietician counsellors) working together to provide both illness care and wellness care respectively and concurrently. The findings also indicated that GPs perceived nurses to be better placed to provide cancer prevention activities. This form of practice will require the development of new relationships between GPs and nurse practitioners that build on their complementary strengths with a clearer focus on which services can be best provided, and by whom [39].

## Limitations

The limitations of self report surveys need to be acknowledged, recognising that it was possible the GPs responded in ways that reflected best practice rather than what they actually do. Also the response rate of 23% needs to be acknowledged. Nevertheless, it can be argued that this does reflect the experience of other research undertaken in primary care and the questionnaires were subjected to power analysis in order to confirm that the level of returns would reasonably reflect the population under study. Other potential limitations associated with the method of data collection also need acknowledge and the potential for the use of electronic questionnaires was considered. However, following some key stakeholder interviews at the start of the study it was it was decided to issue all questionnaires through the Practice Manager in each general practice. Whilst this was undertaken as a measure to increase response rate it needs to be acknowledge that this may have actually reduced the response rate as it is possible the practice manager’s delayed the distribution of the questionnaires.

## Conclusions

Findings from this study indicated that GPs perceive themselves as providing an important role in cancer prevention. This was focused primarily on primary prevention such as smoking cessation within the context of a wider health promotion agenda as well as secondary prevention with the provision of cervical screening. Evidence from this study confirms that GPs are primarily interventionist in their clinical practice and cancer prevention activities are generally opportunistic. While acknowledging that cancer prevention is an integral part of the role of GPs lack of time and remuneration were consistently identified as critical limiting factors. Nevertheless the importance of remuneration as an important barrier for cancer prevention activities in primary care can be questioned as there is a need to balance this with other factors such as practice structure and physician characteristics. The study provided important insights into the potential role of the GP in cancer prevention as seeking to empower and motivate individuals to take responsibility for their own health and make more informed lifestyle choices. It is important to acknowledge, however, that surveys such as this are in large part based on self reporting, and may reflect what GPs think they do or should do. It can be argued that more objective evidence (e.g., chart audits) is needed to see what GPs actually do in practice.

## Competing interests

The authors declare that they have no competing interests.

## Authors’ contributions

SMc contributed to conception and design and drafting of manuscript; SK contributed to conception, design and revising manuscript; HMC contributed to conception, design, interpretation of data and review of manuscript; NMc contributed to data collection, analysis and interpretation of data; GMc contributed to conception and review of manuscript and report. All authors read and approved the final manuscript.

## Pre-publication history

The pre-publication history for this paper can be accessed here:

http://www.biomedcentral.com/1471-2296/14/58/prepub

## Supplementary Material

Additional file 1 Factors affecting the actual and potential role of the General Practitioners in the prevention of cancer. Click here for file
