# Supplementary material for: Investigating the role of the general practitioner in cancer prevention: a mixed methods study
Source: BMC Fam Pract. 2013 May 7;14:58. doi: 10.1186/1471-2296-14-58 (PMC3653692; doi:10.1186/1471-2296-14-58)
Supplement: Additional file 1 — Factors affecting the actual and potential role of the General Practitioners in the prevention of cancer. [file 1471-2296-14-58-S1.doc]

|  |  |
| --- | --- |

**Factors affecting the actual and potential role of the General Practitioners in the prevention of cancer**

**GENERAL PRACTITIONER QUESTIONNAIRE**

In order to minimise the time required to complete the questionnaire, we have presented a series of closed questions that require primarily a ‘Tick Box’ response. It is anticipated that it will take approximately 10 minutes to complete the questionnaire.

Only one box per statement should be ‘ticked’.

**At Section 1, where the response is ‘not at all’, please indicate the reason in the table of options to the right of each page.** (The table at the right side of each page does not require to be completed where a response other than ‘not at all’ has been given).

Should you wish to make any additional comments, please feel free to use the ‘Free-text box’ provided at the end of the questionnaire.

Please feel free to retain a copy of this questionnaire for inclusion in your personal appraisal folder.

Once completed, please return to your Practice Manager.

With thanks for your assistance

**Personal and professional details**

1. **Are you**: Female Male
2. **How many years have you practised as a GP?** ………………..years
3. **Are you**: Full-time Part-time
4. **Are you:**

Principal GP Salaried GP Retained GP Locum GP

1. **Have you Lead Responsibility for Cancer Services within your Practice?**

Yes No

1. **Have you undertaken post-graduate studies in Cancer prevention or treatment?**

Yes No

**Section 1: Actual Role in Cancer Prevention**

| ***Cancer Risk Factor Avoidance-General*** | **Indicate to what extent you personally perform the following activities:**  **(Please Tick)** | | |  | **If you have indicated that you do not perform an activity [Not at all],**  **please indicate the inhibiting factor(s) below:**  **(Please Tick)** | | | | |
| --- | --- | --- | --- | --- | --- | --- | --- | --- | --- |
|  | **Routinely** | **Sometimes** | **Not at all** |  | **Lack of demand** | **Lack of staffing resource** | **Lack of financial resource** | **Lack of time** | **Other**  **(please specify)** |
| Do you provide services relating to cancer prevention for the practice population |  |  |  |  |  |  |  |  |  |
| Do you provide general leaflets/information sheets relating to the prevention of cancer |  |  |  |  |  |  |  |  |  |
| Do you provide general leaflets/information sheets relating to the prevention of cancer in languages other than English |  |  |  |  |  |  |  |  |  |
| Do you provide services relating to cancer prevention specifically designed for patients with special need (eg Learning difficulties) |  |  |  |  |  |  |  |  |  |

| ***Cancer Risk Factor Assessment/Risk Avoidance*** | **Indicate to what extent you personally perform the following activities:**  **(Please Tick)** | | |  | **If you have indicated that you do not perform an activity [Not at all],**  **please indicate the inhibiting factor(s) below:**  **(Please Tick)** | | | | |
| --- | --- | --- | --- | --- | --- | --- | --- | --- | --- |
|  | **Routinely** | **Sometimes** | **Not at all** |  | **Lack of demand** | **Lack of staffing resource** | **Lack of financial resource** | **Lack of time** | **Other**  **(please specify)** |
| **Smoking – Do You:** |  |  |  |  |  |  |  |  |  |
| Enquire about a patient’s smoking habits/history |  |  |  |  |  |  |  |  |  |
| Provide ‘brief advice’ clinics |  |  |  |  |  |  |  |  |  |
| Provide ‘specialist support’ clinics |  |  |  |  |  |  |  |  |  |
| Provide Pharmacotherapy (eg NRT) |  |  |  |  |  |  |  |  |  |
| Provide leaflets/information sheets relating to the dangers of smoking/passive smoking |  |  |  |  |  |  |  |  |  |
| Refer patients to other services |  |  |  |  |  |  |  |  |  |
| **Obesity – Do You:** |  |  |  |  |  |  |  |  |  |
| Measure a patients weight/height/body mass index |  |  |  |  |  |  |  |  |  |
| Provide leaflets/information sheets relating to the relationship between obesity and cancer |  |  |  |  |  |  |  |  |  |
| Display Height/Weight/Body Mass Index Charts in public areas within the Practice |  |  |  |  |  |  |  |  |  |
| Provide ‘weight management’ clinics |  |  |  |  |  |  |  |  |  |
| Refer patients to other services |  |  |  |  |  |  |  |  |  |

| ***Cancer Risk Factor Assessment/Risk Avoidance*** | **Indicate to what extent you personally perform the following activities:**  **(Please Tick)** | | |  | **If you have indicated that you do not perform an activity [Not at all],**  **please indicate the inhibiting factor(s) below:**  **(Please Tick)** | | | | |
| --- | --- | --- | --- | --- | --- | --- | --- | --- | --- |
| ***Cancer Risk Factor Assessment/Risk Avoidance*** | **Indicate to what extent you personally perform the following activities:**  **(Please Tick)** | | |  | **If you have indicated that you do not perform an activity [Not at all],**  **please indicate the inhibiting factor(s) below:**  **(Please Tick)** | | | | |
|  | **Routinely** | **Sometimes** | **Not at all** |  | **Lack of demand** | **Lack of staffing resource** | **Lack of financial resource** | **Lack of time** | **Other**  **(please specify)** |
| **Physical Activity – Do You:** |  |  |  |  |  |  |  |  |  |
| Enquire about a patient’s physical activity levels |  |  |  |  |  |  |  |  |  |
| Provide leaflets/information sheets relating to the requirement for daily physical activity |  |  |  |  |  |  |  |  |  |
| Provide leaflets/information sheets relating to the relationship between physical activity and cancer |  |  |  |  |  |  |  |  |  |
| Refer patients to other services |  |  |  |  |  |  |  |  |  |
| **Diet – Do You:** |  |  |  |  |  |  |  |  |  |
| Enquire about a patient’s diet/eating habits |  |  |  |  |  |  |  |  |  |
| Provide leaflets/information sheets relating to the relationship between diet and cancer eg red meat and processed meat |  |  |  |  |  |  |  |  |  |
| Provide leaflets/information sheets relating to the requirement to consume at least 5 servings of fruit and vegetables daily |  |  |  |  |  |  |  |  |  |
| Refer patients to other services |  |  |  |  |  |  |  |  |  |

| ***Cancer Risk Factor Assessment/Risk Avoidance*** | **Indicate to what extent you personally perform the following activities:**  **(Please Tick)** | | |  | **If you have indicated that you do not perform an activity [Not at all],**  **please indicate the inhibiting factor(s) below:**  **(Please Tick)** | | | | |
| --- | --- | --- | --- | --- | --- | --- | --- | --- | --- |
|  | **Routinely** | **Sometimes** | **Not at all** |  | **Lack of demand** | **Lack of staffing resource** | **Lack of financial resource** | **Lack of time** | **Other**  **(please specify)** |
| **Alcohol – Do You:** |  |  |  |  |  |  |  |  |  |
| Enquire about a patient’s alcohol consumption |  |  |  |  |  |  |  |  |  |
| Provide leaflets/information sheets relating to the consumption of alcohol, identifying (gender-specific) recommended limits of daily alcohol consumption |  |  |  |  |  |  |  |  |  |
| Provide leaflets/information sheets relating to the relationship between alcohol consumption and cancer |  |  |  |  |  |  |  |  |  |
| Refer patients to other services |  |  |  |  |  |  |  |  |  |
| **Exposure to the Sun/UV-Rays – Do You:** |  |  |  |  |  |  |  |  |  |
| Do you:  Enquire about a patient’s potential for sun/UV-Ray exposure |  |  |  |  |  |  |  |  |  |
| Provide leaflets/information sheets relating to the relationship between UV-Ray exposure and cancer |  |  |  |  |  |  |  |  |  |
| Provide information sheets relating to the levels of protection from UV-rays, necessary for fair/sensitive skin |  |  |  |  |  |  |  |  |  |
| Refer patients to other services |  |  |  |  |  |  |  |  |  |
| ***Cancer Risk Factor Assessment/Risk Avoidance*** | **Indicate to what extent you personally perform the following activities:**  **(Please Tick)** | | |  | **If you have indicated that you do not perform an activity [Not at all],**  **please indicate the inhibiting factor(s) below:**  **(Please Tick)** | | | | |
|  | **Routinely** | **Sometimes** | **Not at all** |  | **Lack of demand** | **Lack of staffing resource** | **Lack of financial resource** | **Lack of time** | **Other**  **(please specify)** |
| **Cervical Screening – Do You:** |  |  |  |  |  |  |  |  |  |
| Actively promote cervical screening to all women |  |  |  |  |  |  |  |  |  |
| Provide leaflets/information sheets relating to the benefits of cervical screening and the relationship to cancer |  |  |  |  |  |  |  |  |  |
| Provide cervical screening for all women |  |  |  |  |  |  |  |  |  |
| Refer patients to other services |  |  |  |  |  |  |  |  |  |
| **Other Screening Services – Do You:** |  |  |  |  |  |  |  |  |  |
| Do you:  Actively promote other screening services. If so, please identify below: |  |  |  |  |  |  |  |  |  |
|  |  |  |  |  |  |  |  |  |  |
|  |  |  |  |  |  |  |  |  |  |
|  |  |  |  |  |  |  |  |  |  |
|  |  |  |  |  |  |  |  |  |  |
|  |  |  |  |  |  |  |  |  |  |

**Section 2: Potential Role in Cancer Prevention**

**For each of the following statements please indicate whether you: Strongly agree, Agree, No opinion, Disagree, Strongly disagree**

**(Please Tick):**

| As a GP, I feel my cancer prevention role  ***should be*** *about:* | **Strongly Agree** | **Agree** | **No Opinion** | **Disagree** | **Strongly Disagree** |
| --- | --- | --- | --- | --- | --- |
|  |  |  |  |  |  |
| Empowering individuals to make their own decisions about health issues |  |  |  |  |  |
| Offering advice to inform individuals about better lifestyle choices |  |  |  |  |  |
| Working with local communities to empower them to make decisions about lifestyle choices |  |  |  |  |  |
| Ensuring a coordinated cancer prevention approach within the practice |  |  |  |  |  |
| Identifying patients at risk |  |  |  |  |  |
| Ensuring equality of access to cancer prevention interventions |  |  |  |  |  |

**For each of the following statements please indicate whether you: Strongly agree, Agree, No opinion, Disagree, Strongly disagree**

**(Please Tick):**

| *As a GP, I feel my cancer prevention role*  ***could be*** *developed by:* | ***Strongly Agree*** | ***Agree*** | ***No Opinion*** | ***Disagree*** | ***Strongly Disagree*** |
| --- | --- | --- | --- | --- | --- |
|  |  |  |  |  |  |
| Additional/further inter-professional practice-based training in cancer prevention |  |  |  |  |  |
| Developing the contribution of other health professional staff in strategic planning of cancer prevention within the practice environment |  |  |  |  |  |
| Developing the contribution of other health professional staff in cancer prevention activities within the practice environment |  |  |  |  |  |
| Developing collaboration with other health professionals involved in cancer prevention activities within the practice environment |  |  |  |  |  |
| Developing the nurses’ collaboration with other agencies involved in cancer prevention activities within the community |  |  |  |  |  |
| Developing the nurses’ contribution to cancer prevention activities within the community |  |  |  |  |  |
| Providing additional financial incentives specifically for cancer prevention activities |  |  |  |  |  |
| Providing access to ‘on-line’ resources |  |  |  |  |  |

**Section 3: Attitudes to Cancer Prevention Role**

**For each of the following statements please indicate whether you: Strongly agree, Agree, No opinion, Disagree, Strongly disagree**

**(Please Tick):**

**Self-efficacy of GPs’** in Cancer Prevention

|  | **Strongly Agree** | **Agree** | **No Opinion** | **Disagree** | **Strongly Disagree** |
| --- | --- | --- | --- | --- | --- |
| GPs can motivate patients to live a more healthy lifestyle |  |  |  |  |  |
| GPs plays an important role in Cancer prevention |  |  |  |  |  |
| GPs can contribute to changing patients’ attitude to cancer prevention |  |  |  |  |  |

**Feasibility of GPs’** in Cancer Prevention

|  | **Strongly Agree** | **Agree** | **No Opinion** | **Disagree** | **Strongly Disagree** |
| --- | --- | --- | --- | --- | --- |
| GPs have the time to perform cancer prevention |  |  |  |  |  |
| GPs have the opportunity to perform cancer prevention |  |  |  |  |  |
| Health priorities and targets mitigate against a focussed approach to cancer prevention activity |  |  |  |  |  |

**GPs’ Perceived** Responsibility in Cancer Prevention

|  | **Strongly Agree** | **Agree** | **No Opinion** | **Disagree** | **Strongly Disagree** |
| --- | --- | --- | --- | --- | --- |
| GPs should try and provide cancer prevention |  |  |  |  |  |
| GPs spend too much time on the treatment of cancer rather than providing cancer prevention |  |  |  |  |  |
| GPs have a responsibility to screen high-risk cancer groups |  |  |  |  |  |

**GPs’** Perceived Knowledge:

|  | **Strongly Agree** | **Agree** | **No Opinion** | **Disagree** | **Strongly Disagree** |
| --- | --- | --- | --- | --- | --- |
| I have sufficient knowledge to educate clients about cancer prevention |  |  |  |  |  |
| I require up-to-date information on cancer prevention strategies |  |  |  |  |  |
| I require a better understanding of how to change opinions regarding cancer prevention |  |  |  |  |  |

**GPs’ Perceived** Acceptability to change Cancer Prevention

|  | **Strongly Agree** | **Agree** | **No Opinion** | **Disagree** | **Strongly Disagree** |
| --- | --- | --- | --- | --- | --- |
| Patients are very set in their ways and do not want to change |  |  |  |  |  |
| Patients do not like the GP to meddle in their private life |  |  |  |  |  |
| Patients do not approach their GP for advice on cancer prevention |  |  |  |  |  |
| GPs may increase anxiety in the patient population by undertaking cancer prevention activities |  |  |  |  |  |
| After consultation with a client on cancer risk, I don’t think they will follow my recommendation |  |  |  |  |  |

**Please feel free to add any additional comments you may wish to make in the free-text box below:**

|  |
| --- |
|  |
|  |
|  |
|  |

**You have now completed the questionnaire – Thank you for your assistance. Please return the completed document to your Practice Manager**
